# Two Sides of Quantum-Based Modeling of Enzyme-Catalyzed Reactions: Mechanistic and Electronic Structure Aspects of the Hydrolysis by Glutamate Carboxypeptidase

**DOI:** 10.3390/molecules26206280

**Published:** 2021-10-17

**Authors:** Alexandra V. Krivitskaya, Maria G. Khrenova, Alexander V. Nemukhin

**Affiliations:** 1 Bach Institute of Biochemistry, Federal Research Centre “Fundamentals of Biotechnology” of the Russian Academy of Sciences, 119071 Moscow, Russia; al_krivickaya@mail.ru (A.V.K.); mkhrenova@lcc.chem.msu.ru (M.G.K.); 2 Chemistry Department, M.V. Lomonosov Moscow State University, Leninskie Gory 1/3, 119991 Moscow, Russia; 3 N.M. Emanuel Institute of Biochemical Physics, Russian Academy of Sciences, Kosygina 4, 119334 Moscow, Russia

**Keywords:** reaction mechanism, Gibbs energy profiles, QM/MM molecular dynamics, active site, NBO, QTAIM, protein structure, glutamate carboxypeptidase

## Abstract

We report the results of a computational study of the hydrolysis reaction mechanism of *N*-acetyl-l-aspartyl-l-glutamate (NAAG) catalyzed by glutamate carboxypeptidase II. Analysis of both mechanistic and electronic structure aspects of this multistep reaction is in the focus of this work. In these simulations, model systems are constructed using the relevant crystal structure of the mutated inactive enzyme. After selection of reaction coordinates, the Gibbs energy profiles of elementary steps of the reaction are computed using molecular dynamics simulations with ab initio type QM/MM potentials (QM/MM MD). Energies and forces in the large QM subsystem are estimated in the DFT(PBE0-D3/6-31G**) approximation. The established mechanism includes four elementary steps with the activation energy barriers not exceeding 7 kcal/mol. The models explain the role of point mutations in the enzyme observed in the experimental kinetic studies; namely, the Tyr552Ile substitution disturbs the “oxyanion hole”, and the Glu424Gln replacement increases the distance of the nucleophilic attack. Both issues diminish the substrate activation in the enzyme active site. To quantify the substrate activation, we apply the QTAIM-based approaches and the NBO analysis of dynamic features of the corresponding enzyme-substrate complexes. Analysis of the 2D Laplacian of electron density maps allows one to define structures with the electron density deconcentration on the substrate carbon atom, i.e., at the electrophilic site of reactants. The similar electronic structure element in the NBO approach is a lone vacancy on the carbonyl carbon atom in the reactive species. The electronic structure patterns revealed in the NBO and QTAIM-based analyses consistently clarify the reactivity issues in this system.

## 1. Introduction

Characterization of elementary steps of complex chemical reactions presents one of the major research fields in modern chemistry. A reaction mechanism combines the sequence of molecular events including cleavage and formation of chemical bonds and rearrangement of hydrogen bond networks, etc. In this work, we report results of a computational study of such complex reaction mechanism of an important human enzyme, glutamate carboxypeptidase II (GCPII).

It is hard to overestimate the value of the electronic structure theory to describe these processes, and it is very important to apply the terminology, which is shared by a large community of chemists. In this respect, the Natural Bond Orbital (NBO) analysis developed by Frank Weinhold and his colleagues is an indispensable tool. The most recent papers [1,2] on this subject and references in the latter give an essential understanding of the significance of the NBO analysis. 

Enzyme-catalyzed reactions are literally complex multistep processes that require a careful consideration of each elementary step using adequate instruments. For example, the empirical valence bond theory (EVB) [3,4,5], besides being a practical tool to compute reaction energy profiles, provides a clear picture of electronic structure changes in enzyme active sites. Applications of other electronic structure utilities in enzyme catalysis, including the NBO-based approaches, are presented in the literature as well [6,7,8,9].

When studying enzyme-catalyzed reactions, researchers can emphasize different perspectives to describe their findings, and electronic structures aspects constitute a part of the entire presentation. Experimental works put forward, e.g., kinetic, spectroscopy and crystallography results, whereas computational papers often focus on calculations of the energy profiles, the characterization of reaction intermediates and transition states with the practical goal to interpret experimental data and to make valuable predictions at the mechanistic level. 

**Figure 1:**
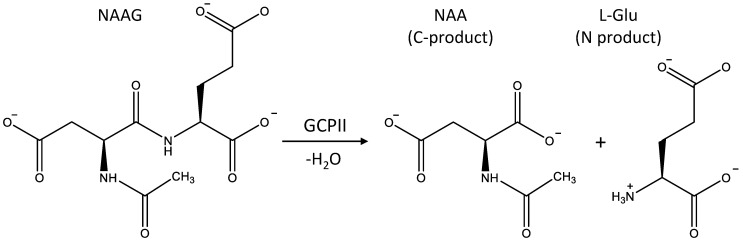
The hydrolysis reaction of *N*-acetyl-l-aspartyl-l-glutamate (NAAG) to the C-product, *N*-acetyl-l-aspartate (NAA), and the N-product, l-glutamate (l-Glu).

We analyze here the reaction mechanism of GCPII, paying attention to both mechanistic and electronic structure aspects. This enzyme is in the public eye, for GCPII is identical to prostate-specific membrane antigen (PSAM), a tumor marker in prostate cancer diagnosis [10]. GCPII is a 750-residue, membrane-associated dinuclear zinc peptidase. The enzyme catalyzes the hydrolysis reaction of *N*-acetyl-l-aspartyl-l-glutamate (NAAG) to *N*-acetyl-l-aspartyl-l-aspartate (NAA) and glutamate, the process occurring in the brain [11]. Figure 1 illustrates the reaction. 

The mechanism of this reaction is not clear enough despite considerable efforts to characterize it in experimental and computational studies. We note several crystal structures of the wild-type and mutated enzymes in the *apo* form as well as with various cofactors [12,13,14,15]. These structures allow one to construct model systems at the atom level, to analyze organization of the active site and to hypothesize the reaction pathway. The key role in the reaction mechanism is often attributed to the Glu424 amino acid residue assuming that this is a catalytic base [12]. This hypothesis is supported by the observation that the Glu424Ala mutation results in complete loss of enzyme activity [14]. 

Refs. [16,17] report results of stationary kinetic measurements of the hydrolysis reactions by GCPII for the wild-type (WT) and the mutated enzymes. In this paper, we use the three-letter designation of amino acids and the single-letter convention to specify protein mutants. For example, the E424A notation refers to the protein in which the Glu424Ala replacement is performed. The V_max_ and k_cat_ values for the WT glutamate carboxypeptidase constitute 101 (96–106) fmol/min [16] and 1.1 ± 0.2 s^−1^ [14], respectively. The latter value of k_cat_ corresponds to the energy barrier of 17.4 kcal/mol, if the transition state theory is applied. Correspondingly, activation barriers at the chemical steps of the entire hydrolysis process should not exceed this amount. Moreover, deposition of quite an extended substrate and release of products (Figure 1) likely refers to the rate-limiting reaction steps. 

In sum, experimental works [12,13,14,15,16,17] provide a considerable amount of structural and kinetic data to be explained in molecular simulations. To the best of our knowledge, Ref. [14] is the only paper that reports the results of quantum mechanics/molecular mechanics (QM/MM) calculations of the potential energy reaction profile for the NAAG hydrolysis by the wild-type GCPII. The authors used the crystal structure PDB ID 2C6C of the enzyme with a tentative transition state analog as a source of atomic coordinates [12]. Two QM-MM partitioning schemes were considered. One with a smaller 175-atomic system was used for geometry optimization, whereas a larger 217-atomic system was applied for single point calculations at the located stationary points. Geometry optimization was performed at the DFT(PBE/def2-SVP) level in QM and single point calculations were carried out at the DFT(B3LYP/def2-TZVP) level. Approximate procedures were applied to estimate transition state structures and to evaluate points on the free energy surfaces. Attempts to locate a configuration of the reaction products were unsuccessful. The obtained reaction profile demonstrates discouraging barrier heights with the energies of 26–29 kcal/mol of the transition states and of the single reaction intermediate above the level of the enzyme-substrate complex. Even if we accept author’s arguments to reduce these amounts by 9 kcal/mol [14], the results of these simulations do not seem convincing. 

**Figure 2:**
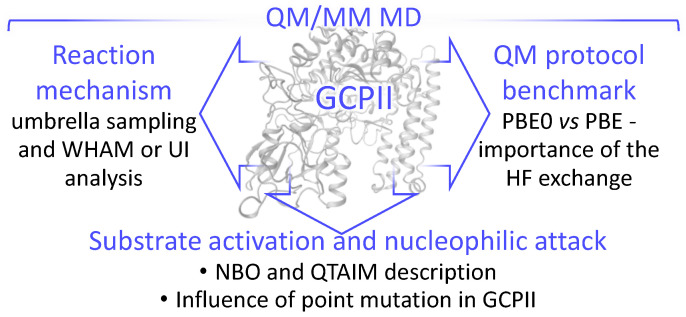
The framework diagram to illustrate this study.

In this work, we apply a more advanced strategy to compute the Gibbs energy profiles for the elementary steps of the title reaction in the wild-type and mutated enzymes (Figure 2). Specifically, we use molecular dynamics simulations with the QM/MM potentials evaluated with the atom-centered basis sets and hybrid functionals. Such QM/MM MD calculations are getting available due to the recent advances in interfacing [18] the MD package NAMD [19,20] with the quantum chemical package TeraChem [21], which is highly efficient on GPUs. It is feasible to apply atom-centered basis sets of 6-31G** or cc-pvdz quality and to use a variety of hybrid functionals in DFT calculations of energies and forces in QM parts and commonly accepted force field parameters in MM parts. When computing MD trajectories of about 10 ps length per window, it is possible to apply the umbrella sampling (US) scan of the Gibbs energy surface with the subsequent statistical analysis, using umbrella integration (UI) methods [22,23] to quantify elementary steps of chemical reactions in the enzyme active sites (see, for example, Refs. [24,25,26,27,28]). Using this technique, we are able to construct reaction energy profiles with realistic barrier heights on the reaction pathway. In our work, we consider two replacements, Glu424Gln and Tyr552Ile, from the available list of point mutations. The E424Q variant reduces V_max_ by 8-fold [16] and the Y552I variant reduces k_cat_ 80-fold [17]. 

Besides simulating mechanistic aspects of this important enzyme-catalyzed reaction, we also focus on the electronic structure details to characterize properties of the reactants (Figure 2). To this goal, we apply the NBO analysis as well as the analysis based on the Laplacian of the electron density, a QTAIM-originated descriptor [29], actively used in many applications including the enzyme catalysis [24,26,28,30,31].

## 2. Models and Methods

The model system containing the substrate NAAG (see Figure 1) in the active site of the WT glutamate carboxypeptidase II was constructed using the crystal structure PDB ID: 3BXM [14] of NAAG complexed with the E424A mutant of GCPII. We substituted Ala424 with the native glutamic acid residue to reconstruct the WT structure. We also constructed structures with single point mutations in the enzyme active site. One of them was the mutation of the catalytic residue leading to the E424Q variant. Another replacement, leading to the Y552I variant, disturbed the oxyanion hole.

**Figure 3:**
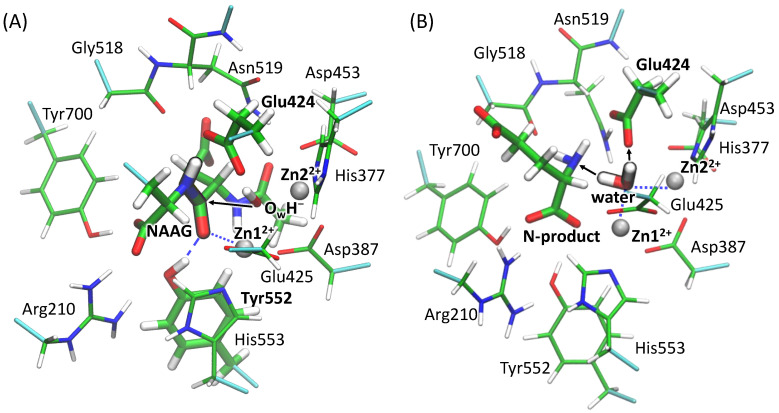
The active site of the wild type GCPII. (**A**) The QM subsystem utilized for the steps 1–4 in the Gibbs energy calculations. The catalytic residue Glu424, the residue Tyr552 that forms the “oxyanion hole”, a catalytic hydroxide anion and the substrate are shown in bold. The peptide fragment to be cleaved in the reaction is highlighted. (**B**) The QM subsystem utilized at the enzyme regeneration step. A water molecule that is responsible for the enzyme regeneration as well as the N-product and Glu424 are highlighted. Color code: zinc—grey, oxygen—red, sulfur—yellow, nitrogen—blue, hydrogen—white, carbon—green. Cyan covalent bonds depict borders between the QM and MM region. Blue dashed and dotted lines are used for the hydrogen and coordination bonds, respectively. Black arrows show the direction of the nucleophilic attack in panel (**A**) and possible proton transfer directions in panel (**B**).

Hydrogen atoms were added using the Reduce program [32] to reproduce the protonation states of amino acids at neutral pH. The model systems were solvated in rectangular water boxes to guarantee that distances from the protein surface to the border of the cell exceeded 12 Å. These systems were neutralized by adding sodium ions. All-atom force fields were utilized; CHARMM36 [33,34] for the protein and TIP3P [35] for water molecules. Systems were preliminary equilibrated during 10 ns in classical MD simulations performed at T = 300 K and *p* = 1 atm with the 1 fs integration time step. The catalytic OH^−^, zinc cations, the side chains of amino acids and the fragment of the substrate forming the coordination bonds with Zn^2+^ ions and the side chain of Glu424 were fixed during this preliminary run. All MD simulations were performed with the NAMD program [19,20]. The RMSD values over all non-hydrogen atoms of the proteins were calculated along the MD trajectories to be sure that the simulation time was enough for the system equilibration. The last frames from the classical MD runs were utilized for the QM/MM MD setup. The QM and MM partitioning is as follows. The QM part includes NAAG and amino acid residues forming hydrogen bonds with the substrate, catalytic hydroxide anion, the catalytic amino acid residue Glu424, zinc ions and their coordination spheres. Coordination spheres of zinc ions are His377, Asp453 for Zn2, Glu425, His553 for Zn1, as well as Asp387 and OH-, which bridges two zinc ions (Figure 3A). We point out the significance of the molecular groups shown in Figure 3. The side chain of Tyr552 forms a hydrogen bond with the oxygen atom of the carbonyl group of the substrate, which contributes to the formation of an “oxyanion hole”; in the Y552I mutant, the QM subsystem includes a water molecule instead of the Tyr552 side chain. The side chains of Tyr700 and Arg210 form hydrogen bonds with the C-terminal carboxyl group of the NAAG. The backbone of Gly518 coordinates the NH fragment, which is significant for the third and fourth reaction steps as described below. The side chain of the glutamate part of NAAG is excluded from the QM subsystem. For the WT model system, the QM part includes 144 atoms described by 1610 basis functions. The QM subsystem discussed above, was considered for the first four steps of reaction. The subsequent enzyme regeneration step was calculated as follows. The C-product, *N*-acetyl-aspartate, was removed from the model system. The space was filled with water molecules. One of them, added to the QM subsystem, was placed between zinc ions (Figure 3B).

**Figure 4:**
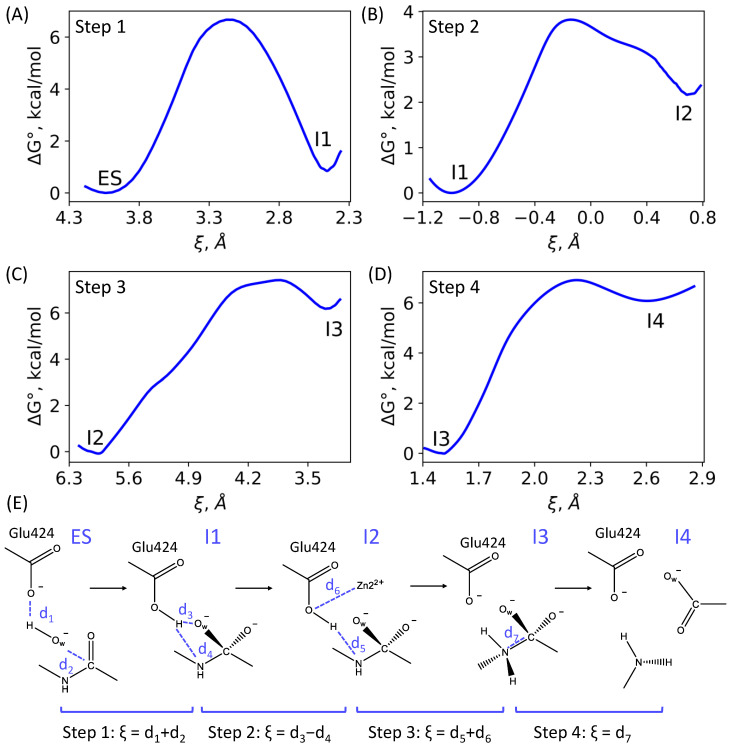
(**A**–**D**) The Gibbs energy profiles of the elementary steps of NAAG hydrolysis in the active site of the wild type GCPII. (**E**) Rearrangements in the active site during each step and reaction coordinates, ξ, utilized in umbrella sampling QM/MM MD simulations.

The TeraChem program [21] was used to calculate forces in the QM region interfaced to the NAMD program according to Ref [18]. The cutoff distance for point charges of the MM subsystem contributing to the QM Hamiltonian was 12 Å. Unconstrained QM/MM MD simulations were performed for 10 ps for each model system. The Gibbs energy profiles for each reaction step along the reaction pathway were calculated using the umbrella sampling approach. The reaction coordinates suggested at each step are shown in Figure 4E. The sets of 5–10 ps runs were performed with harmonic potentials centered at different values of reaction coordinates. The force constant of the harmonic potential ½·K·(ξ − ξ_0_)^2^ was usually set to 40 kcal/mol/Å^2^, and additional trajectories with the K = 80 kcal/mol/Å^2^ in transition state regions were calculated in several runs. The harmonic potentials were centered in every 0.2–0.3 Å segment along the reaction coordinates. The energies and forces in the QM subsystem were calculated at the PBE0 [36] level with D3 dispersion correction [37] for the wild type enzyme and Y552I and E424Q mutants. Calculations were also carried out at the PBE [38] level for the WT enzyme-substrate complex. All QM/MM MD calculations were performed with the 6-31G** basis sets for all atoms except zinc, which was described with the pseudopotentials LANL2DZ. The statistical data analysis was performed using umbrella integration [22,23] approach and verified by the weighted histogram analysis method [39]. 

For the electron density analysis, we selected sets of 50 frames for each model system. The ground state electron density was recalculated at the selected frames, the only difference as compared with the QM/MM MD simulations was that the zinc ions were described with the full-atom 6-31G** basis set. These QM/MM calculations as well as the NBO analysis were performed using the ORCA [40] and NBO 6.0 [41] programs. 

The spatial distributions of the Laplacian of the electron density, ∇^2^*ρ*(**r**), were calculated at different frames of the QM/MM MD trajectory of the ES complex to discriminate reactive and nonreactive species. Previous works [24,30,31,42,43] demonstrate that this approach is a proper tool to visualize the substrate activation in nucleophilic reactions. In molecular systems, the areas of the local electronic charge concentration regions with ∇^2^*ρ*(**r**) < 0 (electrophilic sites) and electronic charge depletion areas with ∇^2^*ρ*(**r**) > 0 (nucleophilic sites) are formed. An analysis of ∇^2^*ρ*(**r**) in the plane formed by the nucleophilic oxygen atom of the catalytic hydroxide anion and the carbon and oxygen atoms of the carbonyl group of the substrate provides easily visible images, which help to discriminate reactive and nonreactive conformations that are favorable and unfavorable for the chemical reaction, respectively. The electron density analysis was performed in the Multiwfn program package [44]. 

## 3. Results

### 3.1. Mechanism of the NAAG Hydrolysis by CGPII

First, we report the results of QM(PBE0-D3/6-31G**)/MM(CHARMM) MD simulations of the entire mechanism of the NAAG hydrolysis in the active site of GCPII. According to our calculations, the reaction mechanism consists of four elementary steps that finally lead to the formation of two products, *N*-acetylaspartate (NAA) and glutamate (see Figure 1 and Figure 4).

The first step is a nucleophilic attack of the hydroxide anion (O_w_H^−^) on the carbonyl carbon atom of NAAG coupled with the proton transfer from O_w_H^−^ to the catalytic Glu424 residue (Figure 4A), leading to the reaction intermediate I1. The reaction coordinate at the first step in the umbrella sampling simulations is the sum of the d(H...O(Glu424)) and d(O_w_...C(NAAG)) distances (Figure 4E). As illustrated in Figure 4A, the activation energy is 6.7 kcal/mol, that is in line with nucleophilic reaction steps calculated for other dinuclear zinc enzymes, metallo-*β*-lactamases [25,45].

The second step describes the conformational change of the carboxylic acid group of the Glu424 side chain leading to the change of its hydrogen bond partner, and inversion of the NH bond of the cleaving peptide fragment of the substrate. Therefore, the reaction coordinate at the second step is the difference between d(H...O_w_) and d(H...N(NAAG)) distances (Figure 4E). In the I1 configuration, the Glu424 –COOH group forms a hydrogen bond with the O_w_ atom, whereas in the configuration of the second reaction intermediate I2, the partner is changed to the nitrogen atom of NAAG. For this reason, the NH group of NAAG changes its hydrogen bond partner from the oxygen atom of Glu424 to the oxygen atom of the Gly518 main chain. This process is required to prepare the NH fragment for the subsequent protonation of nitrogen by a proton of the general base Glu424 (Figure 4B). This stage proceeds with an activation barrier of 4 kcal/mol.

Conformational change in the cleaving peptide fragment during the first and the second reaction steps is an important issue. In the ES configuration, this fragment is slightly disturbed; the HNCO dihedral angle is 156° ± 8°. In the I1 configuration, this angle changes to 163° ± 8°. Together with an inversion of the NH group in the I2 state, the pyramidalization of the nitrogen atom occurs, that is pronounced in the change of the HNCO dihedral angle to −110° ± 12° for the major fraction of this intermediate.

During the third reaction step, the transfer of a proton from Glu424 to the nitrogen atom takes place, and the coordination bond between the oxygen atom of the Glu424 and Zn2 is formed. The reaction coordinate at the third step is the sum of the d(H...N(NAAG) and d(O(Glu424)...Zn2) distances (Figure 4E). This step proceeds with an activation barrier of 7 kcal/mol (Figure 4C). 

The fourth step is the cleavage of the CN bond leading to the formation of separated product species, l-glutamate and *N*-acetyl-l-aspartate (NAA). The reaction coordinate at this stage is the d(C–N) distance (Figure 4E); the computed activation barrier is about 7 kcal/mol (Figure 4D). 

In biochemistry terms, NAA is a C-product and glutamate is an N-product (Figure 1). The latter bears the neutral amino group of the main chain due to the stoichiometry of the reaction. Therefore, we suggest an additional reaction step is required to protonate the amino group at the N-product and simultaneously regenerate the catalytic active site. An initial state of the enzyme assumes the presence of the negatively charged hydroxide anion located between two zinc ions.

**Figure 5:**
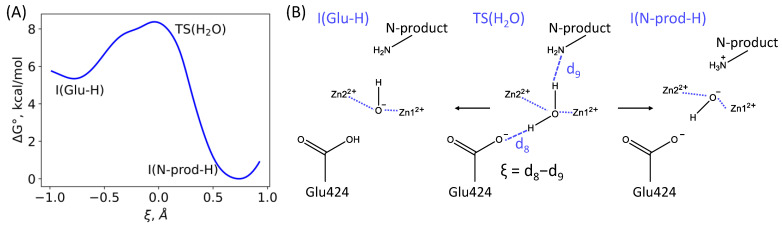
(**A**) The Gibbs energy profile of the enzyme regeneration step. (**B**) Proton transfer pathways at this step. Blue dashed lines are for the distances, d8 and d9, that are included to the reaction coordinate, ξ; blue dotted lines are for the coordination bonds between the water molecule and zinc cations.

NAA is closer to the exit from the active site; therefore, it is reasonable to assume that this species leaves the reaction area in the first place. Correspondingly, we modified the composition of the active site by removing NAA and adding water molecules to fill the space; in particular, a water molecule was placed between the zinc ions. This model system was preliminary equilibrated in classical MD simulations to prevent proton transfer. The subsequent QM/MM MD run resulted in the simultaneous proton transfer from the added water molecule between the zinc ions to the neutral amino group of the N-product. However, it should be noted that there is another proton acceptor in the active site, namely, the catalytic Glu424 residue. We performed umbrella sampling simulations with the reaction coordinate taken as the difference between the H_1_...O(Glu424) and H_2_...N(N-product) distances. The transition state corresponds to the reaction coordinate close to zero, and the analysis of MD trajectories reveals that this configuration corresponds to the state with the water molecule, neutral N-product amino group and negatively charged Glu424 carboxylate. Thus, we formulate an important conclusion, that a water molecule is an unfavorable ligand between two zinc ions, and a proton can be transferred even to the carboxylate species. The computed reaction profile (Figure 5) shows that two channels can be distinguished for deprotonation of the water molecule, namely, the proton is either transferred to the amino group of the N-product or to the Glu424 carboxylate. The N-product acceptor is energetically more favorable resulting in the 8.3 kcal/mol stabilization. The state with the protonated Glu424 is 5.5 kcal/mol higher in energy.

In the following subsections, we concentrate on the first step of the reaction ES → I1 and analyze both mechanistic and electronic structures features, which define catalytic efficiency of this enzyme and its mutants. 

### 3.2. The First Reaction Step: Reactive and Non-Reactive ES Complexes

The nucleophilic attack of the hydroxide anion on the carbonyl carbon atom of NAAG initiates the reaction. From a structural perspective, the substrate is the most flexible species in the enzyme-substrate complex as compared with reaction intermediates occurring at the subsequent reaction steps. Unlike in the latter, the substrate in ES is kept only by non-covalent interactions in the active site. Therefore, a careful analysis of the ES dynamics is important to understand reasons of the observed rate constant changes upon replacement of amino acid residues in the enzyme [24,26,28,46]. 

In our analysis, we consider two mutants of glutamate carboxypeptidase, E424Q and Y552I. In the first case, the catalytic glutamic acid is replaced by the glutamine residue. At the first glance, this should completely abolish the reaction; however, it is known from previous computational studies that glutamine can act as a proton shuttle [27,28,47,48,49]. In fact, performance of the E424Q variant in the NAAG hydrolysis is observed experimentally showing only eight times decrease of the V_max_ value as compared with the wild type enzyme [16]. Another mutation resulting in the Y552I variant leads to 80 times decrease of the rate constant [17]. In the WT enzyme, the OH group of Tyr552 acts as a hydrogen bond donor for the oxygen atom of the substrate carbonyl group, contributing to the so-called “oxyanion hole”. If replaced by a hydrophobic isoleucine residue, the hydrogen bond donor shifts to a water molecule that is more labile in QM/MM MD simulations. 

**Figure 6:**
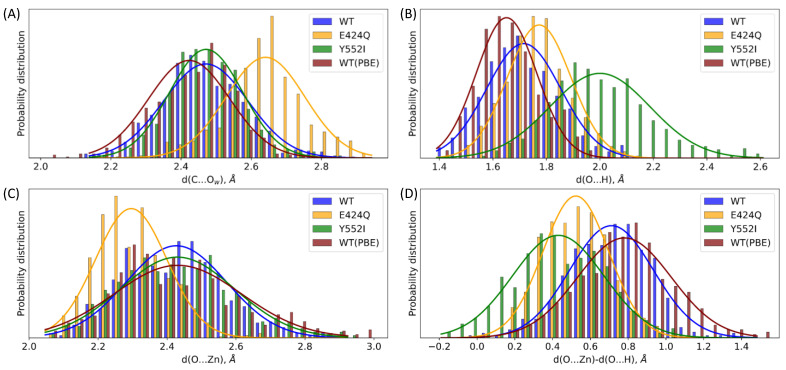
Distributions of distances in the QM/MM MD trajectories of the ES complexes of the wild type (WT, blue) GCPII and its E442Q (orange) and Y552I (green) mutants calculated with the PBE0 functional. Distributions obtained for the WT enzyme computed with the PBE functional are shown in maroon. (**A**) The distance of the nucleophilic attack is (d(C...O_w_); (**B**) hydrogen bond between the oxygen atom of the carbonyl group of the substrate and hydrogen atom of the side chain of Tyr552 (replaced by an auxiliary water molecule in Y552I), d(O...H); (**C**) coordination bond between the oxygen atom of the carbonyl group of the substrate and zinc cation, d(O...Zn); (**D**) difference of d(O...Zn) and d(O...H).

First, we analyze the distributions of the key geometry parameters of the ES complex, namely, the distance of the nucleophilic attack (Figure 6A), the coordination bond (Figure 6C) and the hydrogen bond (Figure 6B), which form the oxyanion hole, and their difference (Figure 6D). The Glu424Gln substitution mostly affects interaction with the catalytic O_w_H^-^ species leading to the pronounced shift of ~0.2 Å of the nucleophilic attack distance distribution to larger values. The distance of the nucleophilic attack observed for all model systems in all QM/MM MD simulations is less than 3 Å. The same features were observed in studies of another dinuclear zinc hydrolase, metallo-*β*-lactamase [25]. Most likely, this is due to a firm fixation of the substrate by the coordination bonds with the metal cations.

The Tyr552Ile point mutation mostly affects the hydrogen bond patterns formed by the carbonyl oxygen of the substrate; the distance distribution is considerably broadened (the standard deviation is increased from 0.14 Å to 0.19 Å), and the average value is 0.3 Å shifted to a longer distance of approximately 2.0 Å. The O...Zn distance distributions were similar for the ES complexes with the wild-type and Y552I enzymes. This distribution for the E424Q species is shifted to smaller values. Combinations of the H...O and O...Zn distances at each MD frame clarify the impact of the oxyanion hole in the catalysis. We find that their differences provide an additional information in this respect as shown in Table 1. 

For the wild type ES complex, the distribution is centered at 0.71 Å, which indicates that the coordination bond is considerably longer than the hydrogen bond. For the less reactive E424Q variant, this value is smaller, being 0.52 Å. Taking together these distributions with the d(O...H) (Figure 6B) and d(O...Zn) distributions (Figure 6C), we conclude that the observed features are due to both elongation of the hydrogen bond and shortage of the coordination bonds. For the Y552I mutant, the distance difference distribution is shifted to even smaller quantities with the mean value of 0.43 Å. Consequently, the qualitative structural measure of the relative catalytic activity might be the distribution of the d(O...Zn) − d(O...H) values calculated at the ES complexes along QM/MM MD trajectories. 

**Figure 7:**
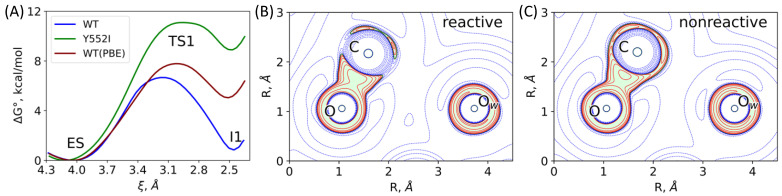
(**A**) The Gibbs energy profiles of the first reaction step calculated for the WT and Y552I GCPII and at the PBE0 level and for the WT enzyme at the PBE level. For the reaction coordinate notation see Figure 4E. (**B**) Reactive and (**C**) nonreactive ES complexes derived from the Laplacian of the electron density analysis in the plane of the carbonyl group of the substrate (C and O atoms) and an oxygen atom, O_w_, of the catalytic O_w_H^−^. Contour lines for the Laplacian of electron density maps are ±(2; 4; 8)∙10^n^ au, −2 ≤ n ≤ 1, blue dashed contour lines indicate the electron density depletion areas (∇^2^*ρ*(**r**) > 0) and red solid lines identify the electron density concentration (∇^2^*ρ*(**r**) < 0), green solid line corresponds to ∇^2^*ρ*(**r**) = 0. The area with ∇^2^*ρ*(**r**) < 0 is colored in light.

To analyze the role of the point mutations in the enzyme, we calculated the corresponding energy profiles at the first reaction step from ES to I1. We intentionally do not consider the E424Q mutant because, in this case, the reaction should proceed via a slightly different pathway due to the change in the proton transfer assisting group, namely, from carboxylate to amide. Figure 7A shows the Gibbs energy profiles calculated for the WT enzyme and for the Y552I mutant. Importantly, the reaction coordinates (d(C...O_w_) + d(H...O)) at the ES minimum energy configuration are similar for both species, being 4.03 Å and 4.12 Å for the WT and Y552I variants, respectively. This is in line with the similar d(C...O_w_) distributions in the WT and Y552I ES complexes (Figure 6A). The differences in profiles are more pronounced, when moving to the transition state region, TS1. In the WT variant, it is narrower, with the maximum at 3.16 Å. Contrary, in the Y552I mutant, this region is flat with the maximum being shifted to 2.96 Å. This is due to a reduced stabilization of the negative charge on the carbonyl oxygen atom in the mutant. Consequently, the first intermediate, I1, is 8 kcal/mol less stable in the mutant as compared with the WT protein. In both cases, the energy barriers are low; therefore, the nucleophilic attack is unlikely the rate-limiting step of the hydrolysis. Thus, we may consider the pre-equilibrium approximation at the first reaction step, and the equilibrium constant between the I1 and ES species modulates the effective rate constant for the entire process. In this particular case, the I1 energy relative to the ES level is important for the reaction rate, but not the TS1 energy.

A more visible analysis of the reactivity of different mutants can be accomplished using an approach based on the Laplacian of electron density, ∇^2^*ρ*(**r**), in the plane of carbonyl group of the substrate (C and O atoms) and the nucleophilic atom, O_w_ [24,30,43]. We extracted a set of 50 frames from each QM/MM MD trajectory of the ES complex. We calculated the 2D map and classified each structure as either reactive or nonreactive. The reactive species (Figure 7B) are characterized by the electron density deconcentration (∇^2^*ρ*(**r**) > 0) in the carbonyl carbon region between the C and O_w_ nuclei. For nonreactive species, the carbonyl carbon atom is enveloped by the electron density concentration region (∇^2^*ρ*(**r**) < 0), (Figure 7C). Table 2 summarizes the results of the 2D ∇^2^*ρ*(**r**) maps analysis. For the WT enzyme, the major fraction of considered structures are reactive (88%). The fraction of reactive species decreases to 6% in the E424Q mutant that is in line with the experimentally observed eight times decrease of the catalytic activity [16]. For Y552I, we found no reactive species among a set of 50 QM/MM MD frames, that is in line with the 80 times decrease of the k_cat_ value compared with the WT enzyme [17]. We conclude that these features of the ES complex are important for the overall catalytic properties of the enzyme. A more efficient substrate activation results in the increase of the effective rate constant, k_cat_. 

To analyze the sensitivity of results to the choice of the functional in DFT-based calculations (see e.g., an important Ref. [50]) we re-calculated properties of the WT model system at the first reaction step (Figure 7A) using the QM(PBE-D3/6-31G**)/MM(CHARMM) approach. We note that dynamical features of the WT ES complexes are similar, if calculated with both PBE0 and PBE functionals in the QM subsystem. Even more, the distribution of the hydrogen bond distances between the oxygen atom of the substrate and Tyr552 OH group is shifted to the smaller values in both approaches (Figure 6B). However, despite similar geometry parameters, strongly different electronic structures calculated at different theory levels may be observed (see e.g., Ref. [24]). Here, we extracted a set of 50 MD frames and calculated 2D maps of the Laplacian of electron density similarly to the strategy described above. Only one of fifty frames calculated at the PBE level in the QM subsystem demonstrated the reactive state. Subsequent calculations of the first step of the reaction show that the position of the ES minimum is essentially the same for both functionals; however, the energy profile calculated at the PBE level demonstrates a worse stabilization of the I1 intermediate. 

### 3.3. The First Reaction Step: The NBO Analysis

The important paper by Glendening and Weinhold [2] presents the natural resonance theory (NRT) analysis for the formamide—formimidic acid tautomerization, exemplifying the simplest reaction with the N-C=O peptide bond. In our study, we deal with a considerably more complex reaction, in which the N-C=O peptide group is involved (see Figure 1). We remind that a nucleophilic attack of the hydroxide anion on the carbon atom in this group takes place at the first reaction step of the enzyme-catalyzed reaction. Correspondingly, the reactivity of this fragment is an important issue. It should be noted that we failed to apply the NRT analysis in the straightforward fashion to evaluate relative weights of various resonant electronic structures due to the large size of the model system, which requires more than 1600 basis functions in the QM subsystem. Therefore, we apply here another strategy. We analyze a set of frames extracted from QM/MM MD trajectories of the ES complexes of the wild-type glutamate carboxypeptidase and its E424Q and Y552I mutants. Specifically, 50 frames are selected for every trajectory. We focus on the NBO features revealed in the electronic structure analysis for each frame and count how many different structures occur in the set. 

**Figure 8:**
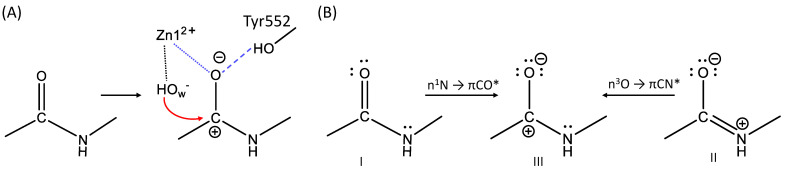
(**A**) The substrate activation in the active site of the enzyme. Dotted lines indicate the coordination bonds and the dashed line indicates a hydrogen bond. Blue lines show the interaction between the substrate and the “oxyanion hole”; the red arrow shows a direction of the nucleophilic attack. (**B**) Structures found by the NBO analysis of the ES complexes.

Figure 8 and Table 3 illustrates the major findings of this analysis. We assume that structure III in panel (B) is the most reactive one. In this structure, the bond between C and O atoms is single; three lone pairs are localized on the oxygen atom making it strongly negative; a free valence (LV, or lone valence according to the NBO classification) is formed on the carbon atom, which should facilitate the nucleophilic attack of the hydroxide anion. We also suppose that structure I is the least reactive one, since it has neither a vacant carbon valence, nor a third lone electron pair on the oxygen atom. Structure II, accordingly, occupies an intermediate position. 

These three electronic structures in the wild-type ES complex were identified with a I:II:III ratio of 18:31:1 for the PBE0 calculation. Only I and II types of structures are found in both mutants in ratios 10:40 and 36:14 for the E424Q and Y552I mutants, respectively. The MD frames with the NBO structure III are not observed in mutated complexes, thus explaining a lower catalytic activity observed in the experiments [16,17]. The mutant E424Q has a higher catalytic activity as compared to the Y552I variant, which is reflected in the ratio of NBO structures I and II: the structure II is predominant in E424Q, whereas the less reactive I structure is the major component in Y552I. 

We also note that the use of the PBE functional in calculations with the WT enzyme shows the ratio 8:42:1. 

We think that consideration of the electronic structure patterns revealed in the NBO analysis, e.g., those shown in Figure 8B, assists in the design of models in semi-empirical approaches, in particular, to recognize the necessary components for the empirical valence bond (EVB) applications [3,4,5].

It is interesting to compare results of the second order perturbation theory analysis of the Fock matrix in the NBO basis, which provides stabilization energies of the interaction between different NBO patterns, accounting for the presence of the minor resonance state and their population [2]. The n_N_ → π_CO_* interactions account for the delocalization between structures I and III, whereas the n_O_ → π_CN_* interactions quantify delocalization between structures II and III (Table 4). In wild type ES, the interactions of both types are more pronounced than in the mutated structures. The same issue is noted for the WT enzyme calculated at the PBE level. In other words, there is a larger contribution of the reactive III pattern in the structures, in with the I and II patterns dominate. This is in line with the results of the Laplacian of electron density analysis that a higher degree of the substrate activation in the active site of the WT enzyme is noted as compared with that in the mutants. 

## 4. Conclusions

The title of the paper promises the consideration of two sides of modeling a specific enzyme-catalyzed multistep chemical reaction. The mechanistic aspects of the hydrolysis of *N*-acetyl-l-aspartyl-l-glutamate by glutamate carboxypeptidase as revealed in our simulations are described in Section 3.1 and partly in Section 3.2. Specifically, the computed Gibbs energy profiles of each reaction step exhaustively determine the entire mechanism of this reaction. These findings constitute the primary results to be reported in computational simulations of enzyme catalysis. We emphasize that our models explain the role of point mutations in the enzyme observed in the experimental kinetic studies. We do not pay much attention here to the structural issues, but note that the computational results well agree with the results of crystallography data, as in many of present-day DFT-based QM/MM simulations of active site structures in enzymes. The established reaction mechanism here improves some shortcomings of the previous simulations of the GCPII catalysis described in the computational part of the paper [14]. We should note that the use of the QM(DFT)/MM MD approach with the atom-centered basis sets and reliable hybrid functionals in QM-subsystems allows one to make an essential step forward in calculations of the Gibbs energy profiles of complex chemical reactions in proteins.

Another side of the present study refers to the electronic structure analysis of the critical fragment of the reaction pathway, namely that from the ES complex to the first reaction intermediate. We apply here both QTAIM-based approaches (2D maps of the Laplacian of electron density) and the NBO analysis. The results of this consideration illuminate the qualitative aspects of reactivity, which are important for the development of concepts in the enzyme catalysis. In the mutated enzyme, the reaction rate constants are lower than those in the wild-type enzyme due to diminishing substrate activation as observed consistently in the NBO analysis and in the Laplacian of electron density maps. 

## Figures and Tables

**Table 1:** Distributions of key interatomic distances from QM/MM MD trajectories at the ES configuration for the WT, E424Q and Y552I species calculated at the PBE0 level and for the ES WT complex calculated at the PBE level. The distances are in Å.

Geometry Parameter	WT	E424Q	Y552I	WT (PBE)
d(C...Ow)	2.47 ± 0.12	2.64 ± 0.11	2.47 ± 0.10	2.42 ± 0.12
d(O...H)	1.72 ± 0.14	1.77 ± 0.12	2.00 ± 0.19	1.65 ± 0.11
d(O...Zn)	2.43 ± 0.15	2.30 ± 0.10	2.43 ± 0.17	2.43 ± 0.18
d(O...Zn) − d(O...H)	0.71 ± 0.22	0.52 ± 0.17	0.43 ± 0.24	0.78 ± 0.25
d(O...Zn) + d(O...H)	4.14 ± 0.18	4.07 ± 0.14	4.43 ± 0.26	4.08 ± 0.18

**Table 2:** Fractions of the reactive species obtained in the analysis of the Laplacian of electron density of the sets of 50 frames from QM/MM MD trajectories at the ES configuration for the WT, E424Q and Y552I species calculated at the PBE0 level and for the ES WT complex calculated at the PBE level.

Reactivity	WT	E424Q	Y552I	WT (PBE)
Fraction of reactive conformations	88%	6%	0%	2%

**Table 3:** Fractions of the NBO structures of types I, II and III (see Figure 8 for further details) obtained from the sets of 50 frames along QM/MM MD trajectories at the ES configuration for the WT, E424Q and Y552I species calculated at the PBE0 level and the ES WT complex calculated at the PBE level.

Structure Type	WT	E424Q	Y552I	WT (PBE)
I	36%	20%	72%	16%
II	62%	80%	28%	82%
III	2%	0%	0%	2%

**Table 4:** Average values of the second order perturbation theory estimates of the interaction strength for types I and II structures for the WT GCPII, its E424Q and Y552I mutants calculated at the PBE0 level, and for the WT enzyme calculated at the PBE level. The energies (kcal/mol) account for the extent of delocalization between either I or II type structures and the structure III, which is considered as the most reactive one (see Figure 7B).

Structure Type	Interaction Type	WT	E424Q	Y552I	WT (PBE)
I	n_N_ → π_CO_*	63	33	36	34
II	n_O_ → π_CN_*	47	24	26	21

## Data Availability

The structures corresponding to the frames near the reaction intermediates are deposited to the general-purpose open-access repository ZENODO and can be accessed via https://doi.org/10.5281/zenodo.5513308 (accessed on 12 October 2021).

## Sample Availability

Samples of the compounds are not available from the authors.

## References

[1] Weinhold F. (2021). The Path to Natural Bond Orbitals. Isr. J. Chem..

[2] Glendening E.D., Weinhold F. (2021). Pauling’s Conceptions of Hybridization and Resonance in Modern Quantum Chemistry. Molecules.

[3] Kamerlin S.C.L., Warshel A. (2011). The empirical valence bond model: Theory and applications. WIREs Comput. Mol. Sci..

[4] Kamerlin S.C.L., Warshel A. (2010). The EVB as a quantitative tool for formulating simulations and analyzing biological and chemical reactions. Faraday Discuss..

[5] Åqvist J., Warshel A. (1993). Simulation of enzyme reactions using valence bond force fields and other hybrid quantum/classical approaches. Chem. Rev..

[6] Lever G., Cole D.J., Lonsdale R., Ranaghan K.E., Wales D.J., Mulholland A.J., Skylaris C.-K., Payne M.C. (2014). Large-Scale Density Functional Theory Transition State Searching in Enzymes. J. Phys. Chem. Lett..

[7] Prejanò M., Marino T., Russo N. (2018). QM Cluster or QM/MM in Computational Enzymology: The Test Case of LigW-Decarboxylase. Front. Chem..

[8] Prejanò M., Alberto M.E., Russo N., Toscano M., Marino T. (2020). The Effects of the Metal Ion Substitution into the Active Site of Metalloenzymes: A Theoretical Insight on Some Selected Cases. Catalysts.

[9] Stuyver T., De Proft F., Geerlings P., Shaik S. (2020). How Do Local Reactivity Descriptors Shape the Potential Energy Surface Associated with Chemical Reactions? The Valence Bond Delocalization Perspective. J. Am. Chem. Soc..

[10] Su S.L., Huang I.P., Fair W.R., Powell C.T., Heston W.D. (1995). Alternatively spliced variants of prostate-specific membrane antigen RNA: Ratio of expression as a potential measurement of progression. Cancer Res..

[11] Robinson M.B., Blakely R.D., Couto R., Coyle J.T. (1987). Hydrolysis of the brain dipeptide N-acetyl-L-aspartyl-L-glutamate. Identification and characterization of a novel N-acetylated alpha-linked acidic dipeptidase activity from rat brain. J. Biol. Chem..

[12] Mesters J.R., Barinka C., Li W., Tsukamoto T., Majer P., Slusher B.S., Konvalinka J., Hilgenfeld R. (2006). Structure of glutamate carboxypeptidase II, a drug target in neuronal damage and prostate cancer. EMBO J..

[13] Barinka C., Hlouchova K., Rovenska M., Majer P., Dauter M., Hin N., Ko Y.-S., Tsukamoto T., Slusher B.S., Konvalinka J. (2008). Structural basis of interactions between human glutamate carboxypeptidase II and its substrate analogs. J. Mol. Biol..

[14] Klusák V., Bařinka C., Plechanovová A., Mlčochová P., Konvalinka J., Rulíšek L., Lubkowski J. (2009). Reaction Mechanism of Glutamate Carboxypeptidase II Revealed by Mutagenesis, X-ray Crystallography, and Computational Methods. Biochemistry.

[15] Navrátil M., Tykvart J., Schimer J., Pachl P., Navrátil V., Rokob T.A., Hlouchová K., Rulíšek L., Konvalinka J. (2016). Comparison of human glutamate carboxypeptidases II and III reveals their divergent substrate specificities. FEBS J..

[16] Speno H.S., Luthi-Carter R., Macias W.L., Valentine S.L., Joshi A.R., Coyle J.T. (1999). Site-directed mutagenesis of predicted active site residues in glutamate carboxypeptidase II. Mol. Pharmacol..

[17] Mlcochová P., Plechanovová A., Barinka C., Mahadevan D., Saldanha J.W., Rulísek L., Konvalinka J. (2007). Mapping of the active site of glutamate carboxypeptidase II by site-directed mutagenesis. FEBS J..

[18] Melo M.C.R., Bernardi R.C., Rudack T., Scheurer M., Riplinger C., Phillips J.C., Maia J.D.C., Rocha G.B., Ribeiro J.V., Stone J.E. (2018). NAMD goes quantum: An integrative suite for QM/MM simulations. Nat. Methods.

[19] Phillips J.C., Hardy D.J., Maia J.D.C., Stone J.E., Ribeiro J.V., Bernardi R.C., Buch R., Fiorin G., Hénin J., Jiang W. (2020). Scalable molecular dynamics on CPU and GPU architectures with NAMD. J. Chem. Phys..

[20] Phillips J.C., Braun R., Wang W., Gumbart J., Tajkhorshid E., Villa E., Chipot C., Skeel R.D., Kalé L., Schulten K. (2005). Scalable molecular dynamics with NAMD. J. Comput. Chem..

[21] Seritan S., Bannwarth C., Fales B.S., Hohenstein E.G., Isborn C.M., Kokkila-Schumacher S.I.L., Li X., Liu F., Luehr N., Snyder J.W. (2020). TeraChem: A graphical processing unit-accelerated electronic structure package for large-scale ab initio molecular dynamics. WIREs Comput. Mol. Sci..

[22] Kästner J. (2011). Umbrella sampling. Wiley Interdiscip. Rev. Comput. Mol. Sci..

[23] Kästner J., Thiel W. (2005). Bridging the gap between thermodynamic integration and umbrella sampling provides a novel analysis method: “Umbrella integration”. J. Chem. Phys..

[24] Khrenova M.G., Tsirelson V.G., Nemukhin A.V. (2020). Dynamical properties of enzyme–substrate complexes disclose substrate specificity of the SARS-CoV-2 main protease as characterized by the electron density descriptors. Phys. Chem. Chem. Phys..

[25] Krivitskaya A.V., Khrenova M.G. (2021). Boronic Acids as Prospective Inhibitors of Metallo-β-Lactamases: Efficient Chemical Reaction in the Enzymatic Active Site Revealed by Molecular Modeling. Molecules.

[26] Khrenova M.G., Kulakova A.M., Nemukhin A.V. (2021). Light-Induced Change of Arginine Conformation Modulates the Rate of Adenosine Triphosphate to Cyclic Adenosine Monophosphate Conversion in the Optogenetic System Containing Photoactivated Adenylyl Cyclase. J. Chem. Inf. Model..

[27] Khrenova M.G., Bulavko E.S., Mulashkin F.D., Nemukhin A.V. (2021). Mechanism of Guanosine Triphosphate Hydrolysis by the Visual Proteins Arl3-RP2: Free Energy Reaction Profiles Computed with Ab Initio Type QM/MM Potentials. Molecules.

[28] Khrenova M.G., Grigorenko B.L., Nemukhin A.V. (2021). Molecular Modeling Reveals the Mechanism of Ran-RanGAP-Catalyzed Guanosine Triphosphate Hydrolysis without an Arginine Finger. ACS Catal..

[29] Bader R.F.W. (1991). A quantum theory of molecular structure and its applications. Chem. Rev..

[30] Carroll M.T., Cheeseman J.R., Osman R., Weinstein H. (1989). Nucleophilic addition to activated double bonds: Predictions of reactivity from the Laplacian of the charge density. J. Phys. Chem..

[31] Khrenova M.G., Krivitskaya A.V., Tsirelson V.G. (2019). The QM/MM-QTAIM approach reveals the nature of the different reactivity of cephalosporins in the active site of L1 metallo-β-lactamase. New J. Chem..

[32] Word J.M., Lovell S.C., Richardson J.S., Richardson D.C. (1999). Asparagine and glutamine: Using hydrogen atom contacts in the choice of side-chain amide orientation. J. Mol. Biol..

[33] Best R.B., Zhu X., Shim J., Lopes P.E.M., Mittal J., Feig M., MacKerell A.D. (2012). Optimization of the Additive CHARMM All-Atom Protein Force Field Targeting Improved Sampling of the Backbone ϕ, ψ and Side-Chain χ_1_ and χ_2_ Dihedral Angles. J. Chem. Theory Comput..

[34] Denning E.J., Priyakumar U.D., Nilsson L., Mackerell A.D. (2011). Impact of 2′-hydroxyl sampling on the conformational properties of RNA: Update of the CHARMM all-atom additive force field for RNA. J. Comput. Chem..

[35] Jorgensen W.L., Chandrasekhar J., Madura J.D., Impey R.W., Klein M.L. (1983). Comparison of simple potential functions for simulating liquid water. J. Chem. Phys..

[36] Adamo C., Barone V. (1999). Toward reliable density functional methods without adjustable parameters: The PBE0 model. J. Chem. Phys..

[37] Grimme S., Antony J., Ehrlich S., Krieg H. (2010). A consistent and accurate ab initio parametrization of density functional dispersion correction (DFT-D) for the 94 elements H-Pu. J. Chem. Phys..

[38] Perdew J.P., Burke K., Ernzerhof M. (1996). Generalized Gradient Approximation Made Simple. Phys. Rev. Lett..

[39] Grossfield A. WHAM: The Weighted Histogram Analysis Method. http://membrane.urmc.rochester.edu/content/wham.

[40] Neese F. (2012). The ORCA program system. Wiley Interdiscip. Rev. Comput. Mol. Sci..

[41] Glendening E.D., Landis C.R., Weinhold F. (2013). NBO 6.0: Natural bond orbital analysis program. J. Comput. Chem..

[42] Khrenova M.G., Kulakova A.M., Nemukhin A.V. (2020). Proof of concept for poor inhibitor binding and efficient formation of covalent adducts of KRAS G12C and ARS compounds. Org. Biomol. Chem..

[43] Khrenova M.G., Nemukhin A.V., Tsirelson V.G. (2020). Discrimination of enzyme–substrate complexes by reactivity using the electron density analysis: Peptide bond hydrolysis by the matrix metalloproteinase-2. Mendeleev Commun..

[44] Lu T., Chen F. (2012). Multiwfn: A multifunctional wavefunction analyzer. J. Comput. Chem..

[45] Khrenova M.G., Nemukhin A.V. (2018). Modeling the Transient Kinetics of the L1 Metallo-β-Lactamase. J. Phys. Chem. B.

[46] Khrenova M.G., Tsirelson V.G., Nemukhin A.V. (2020). Computational Characterization of the Substrate Activation in the Active Site of SARS-CoV-2 Main Protease. Supercomput. Front. Innov..

[47] Grigorenko B.L., Kots E.D., Nemukhin A.V. (2019). Diversity of mechanisms in Ras–GAP catalysis of guanosine triphosphate hydrolysis revealed by molecular modeling. Org. Biomol. Chem..

[48] Khrenova M.G., Grigorenko B.L., Kolomeisky A.B., Nemukhin A.V. (2015). Hydrolysis of guanosine triphosphate (GTP) by the Ras-GAP protein complex: Reaction mechanism and kinetic scheme. J. Phys. Chem. B.

[49] Khrenova M.G., Nemukhin A.V., Domratcheva T. (2013). Photoinduced electron transfer facilitates tautomerization of the conserved signaling glutamine side chain in BLUF protein light sensors. J. Phys. Chem. B.

[50] Mardirossian N., Head-Gordon M. (2017). Thirty years of density functional theory in computational chemistry: An overview and extensive assessment of 200 density functionals. Mol. Phys..

